# Assessment of the Measurement Performance of the Multimodal Fibre Optic Shape Sensing Configuration for a Morphing Wing Section

**DOI:** 10.3390/s22062210

**Published:** 2022-03-12

**Authors:** Nakash Nazeer, Roger M. Groves, Rinze Benedictus

**Affiliations:** 1 Aerospace NDT Laboratory, Faculty of Aerospace Engineering, Delft University of Technology, Kluyverweg 1, 2629 HS Delft, The Netherlands; r.m.groves@tudelft.nl; 2 Structural Integrity & Composites, Faculty of Aerospace Engineering, Delft University of Technology, Kluyverweg 1, 2629 HS Delft, The Netherlands; r.benedictus@tudelft.nl

**Keywords:** Fibre Bragg Grating, strain measurement, optical interferometry, spectral sensing, shape sensing, structural health monitoring, morphing wing, experimental mechanics, multimodal sensing, optical fibre sensing

## Abstract

In this paper, with the final aim of shape sensing for a morphing aircraft wing section, a developed multimodal shape sensing system is analysed. We utilise the method of interrogating a morphing wing section based on the principles of both hybrid interferometry and Fibre Bragg Grating (FBG) spectral sensing described in our previous work. The focus of this work is to assess the measurement performance and analyse the errors in the shape sensing system. This includes an estimation of the bending and torsional deformations of an aluminium mock-up section due to static loading that imitates the behaviour of a morphing wing trailing edge. The analysis involves using a detailed calibration procedure and a multimodal sensing algorithm to measure the deflection and shape. The method described In this paper, uses a standard single core optical fibre and two grating pairs on both the top and bottom surfaces of the morphing section. A study on the fibre placement and recommendations for efficient monitoring is also included. The analysis yielded a maximum deflection sensing error of 0.7 mm for a 347 × 350 mm wing section.

## 1. Introduction

Morphing capable aircraft structures have seen a rise in the aerospace field due to the advent of smart materials and structures [1]. An interesting and important feature of these structures is the monitoring of the shape of their continuously deforming sections. Deflection monitoring is a crucial parameter used as an index for shape sensing [2].

Shape sensing can alternatively be defined as the process of using data from discrete strain measurements to reconstruct the displacement field of a structure [3]. With regard to morphing wings, shape sensing aids in regulating and monitoring the in-flight loads [4] as well as in the optimisation and the increase in aerodynamic efficiency in flight [5].

Morphing structures are required to be rigid as well as flexible at the same time to carry out the morphing sequences. This is particularly challenging for the morphing design as it must be capable of resisting high in-plane strains whilst also having low in-plain stiffnesses. Moreover, an adequate amount of out-of-plane stiffness would also be required to resist the aerodynamic loads as well as local buckling [6]. For a complex system such as this, monitoring the shape plays a key role in understanding the behaviour of the structure and also to make sure it is operating within its design limit. This information can also be used to track the movements of the structure and hence estimate its shape through out the morphing sequence.

For shape sensing, visual (camera-based) sensing methods are not preferred as they require the processing of large amounts of data and are not practical to be installed on a flying aircraft [7]. The most common method used nowadays for shape sensing is based on surface attachable sensors that map displacements based on strain data [8,9]. The objective is to have sensors that can be fitted on or in an aircraft wing and to fly with them.

Shape sensing using optical fibres have been widely used as a health monitoring strategy in various fields including aerospace [2,3], civil [10] and medical [11] applications. Although different sensing methods such as piezoelectric sensing [12] and fringe projection [13] can be adopted for the same purpose, they do not fare well when compared to optical fibres considering EMI immunity, resistance to corrosion, high sensitivity and unobtrusive nature when bonded on or embedded in a structure [14,15]. Fibre Bragg Grating (FBG) based optical fibre sensing is the most favoured technology for shape sensing [16] and for the monitoring of aircraft wing deformations [17,18]. The National Aeronautics and Space Administration (NASA) famously reported the shape sensing of an aircraft wing under bending loads. The system contained a series of FBGs bonded at specific points along the span of the wing. Multiple strain measurements were taken on the ground and during flight and were used to reconstruct the shape of the wing [19,20].

It can be seen that the previous works primarily focus on actuation, morphing mechanism and sensor design. Our work, on the other hand, mainly focusses on simultaneously monitoring these morphing movements for shape sensing and load monitoring purposes. This study presents a shape sensing method capable of handling the geometrical complexities of a seamless wing trailing edge morphing concept. Additionally, a claim is also made for the optimum location for the fibre on a morphing section.

In this paper, we investigate the deformed shape estimation using optical fibre technology with a quasi-distributed Fibre Bragg Grating (FBG) sensor layout. This work is an extension of the successful demonstration of the potential of this method on a 1-dimensional case involving a cantilever beam [21], a 2-dimensional load monitoring study on a cantilever plate [22] and in a composite wing prototype [23]. A morphing wing-section model (with similar shape to the SmartX prototype) is used for the experimental study. There are 3 research questions established for this work and they are as follows: (a) How can the multimodal sensing approach be used for the monitoring of a complex morphing wing section? (b) What is the preferred fibre location for effective monitoring and differentiating between bend up, bend down, twist and their combinations? and (c) How effective is the multimodal method in estimating the tip deflection during morphing? In this paper, we will demonstrate that with 2 FBG-Pairs forming an arrangement similar to a long Fabry–Pérot configuration (forming an FBG-Pair) the deformed shape of the structure can be determined. An experimental based model is used to determine arbitrary deformed shapes.

This also includes the optimum location for the fibres for the monitoring. effectiveness, accuracy and error estimation of morphing deflection sensing.

This paper is organised as follows. A brief introduction of the focus of this study is summarised in Section 1 with a focus on optical fibre sensing. Section 2 very briefly (and in detail in [22]) presents the theory of the hybrid optical sensing method used for shape sensing. Section 3 explains the setup including the morphing trailing edge geometry, sensor design and the optical fibre layout. Section 4 introduces the different morphing cases, the calibration procedure, arbitrary morphing sequences and the algorithm. The results are compiled in Section 5, whilst Section 6 discusses these findings. Finally, the conclusions are summarised in Section 7.

## 2. Theory

The multimodal shape sensing principle has been described in detail previously [22]. Here, we concisely provide the basic theory.

The optical fibre shape sensing approach incorporated in this study materialises from the combination of spectral sensing (Section 2.1) for local FBG sensor measurement and interferometric sensing (Section 2.2) for measuring between FBG sensor pairs.

### 2.1. FBG Spectral Sensing

Fibre Bragg gratings (FBGs) are periodic/aperiodic modulations in the refractive index of a fibre induced by laterally exposing its core with a UV laser [24]. These gratings behave as a wavelength selective mirror that acts as a spectral bandpass filter in reflection and a bandstop filter in transmission.

When the grating period (Λ) is about half the wavelength of the incident light, by successive coherent scattering, only light within a narrow spectral width centred at the Bragg wavelength ($\lambda_{B}$) [25] is reflected. As a result of external mechanical interferences there is a shift in the Bragg wavelength ($\Delta \lambda_{B}$) which can be expressed in the form of strain. This change in calculated strain (ε) is expressed as the shift in Bragg wavelength [26].

**Figure 1:**
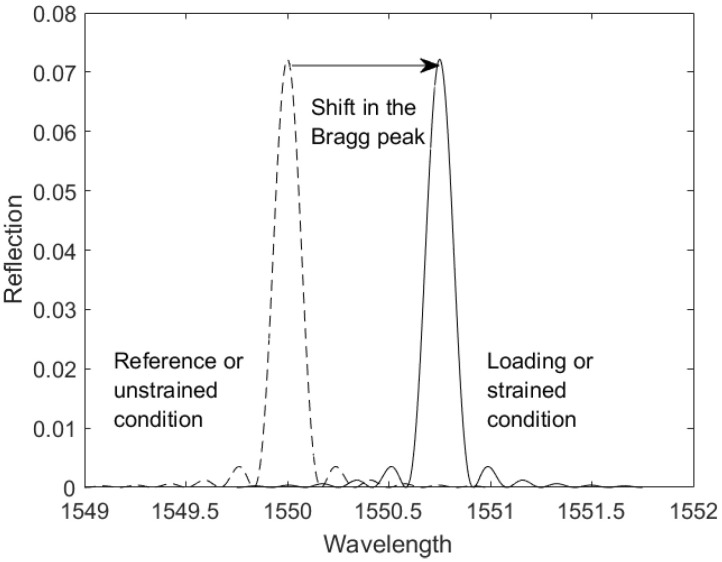
Shift in the Bragg peak ($\Delta \lambda_{B}$) due to an applied strain.

An example spectra obtained of an FBG peak is as shown in Figure 1.

### 2.2. FBG-Pair Interferometric Sensing

The FBG-Pair interferometric sensing involves two FBGs that form a region similar to a long Fabry–Pérot cavity [27]. The measurement is done by comparing the phase differences of interference patterns between two FBGs. The phase difference is a function of the length of the cavity (ΔL) and is altered in the presence of external mechanical disturbances. In order to analyse the change in interference patterns, a reference fibre is used containing a mirror at its end. The fibre that contains this sensing cavity is referred henceforth as the ”sensing fibre”.

**Figure 2:**
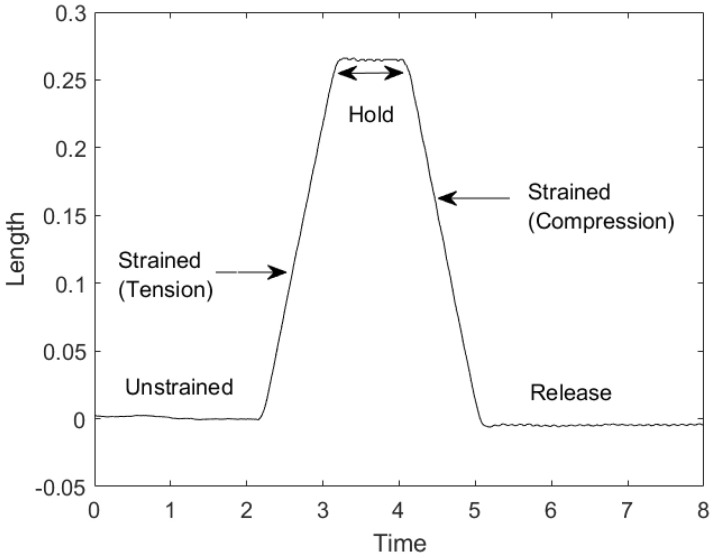
Change in path-length (ΔL) between two given Fibre Bragg Gratings (FBG’s) due to an applied strain.

An example spectra obtained of an FBG-Pair is as shown in Figure 2. The effect of tension and compression on the region between two FBGs is depicted.

### 2.3. Multimodal Sensing Principle

The principle of this sensing method is based on a two-step FBG measurement procedure of spectral sensing (Section 2.1) of each grating sensor combined with interferometric sensing (Section 2.2) between grating sensor pairs.

Data acquisition from the two mentioned sensing methods is required for this measurement procedure. The local strain (ε) at each FBG is calculated by analysing their spectral shifts. Simultaneously, the change in optical path-length (ΔL) between any two given FBGs is calculated by measuring the displacement in terms of strain. Since grating ‘pairs’ are required in the multimodal sensing, the minimum number of FBGs required is always an even number. The total number of pairs however vary with application.

This gives two sets of measurements. The first is taken from local strain readings of each FBG, denoted as $\varepsilon_{1-n}$, where *n* = total number of FBGs. The second set is from the interferometric measurements between two FBGs, denoted as $\Delta L_{i-j}$, where *i* and *j* are FBG identifiers. The stepwise execution of the two measurement groups to estimate the shape is explained in detail in the methodology section (Section 4).

## 3. Methodology

The test specimen is inspired from the wing warping/bending concept [28] and is a construction of this concept sections with a focus on its morphing mechanism.

**Figure 3:**
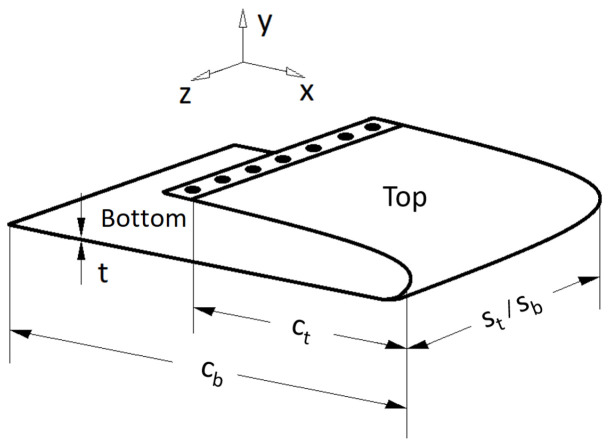
Geometry and coordinate system of the test wing section.

The geometry and coordinate system of the specimen used is as shown in Figure 3 along with the nomenclature used. The test specimen is a pseudo wing-section consisting of two parts that are referred to as the top section and the bottom section. These two surfaces are part of a single construction formed by bending an aluminium sheet over an actual conventional wing section. The geometric properties of the wing are as shown in Table 1. The top and bottom sections of the wing have a chord *c*, span *s*, and thickness *t*. The fixed and free ends of the wing section are denoted further on as the root and the tip, respectively. The deflection happens in the y-axis which varies along the chord depending on the actuation and the morphing sequence. This wing section will be hereon referred to simply as the ”wing”.

The wing undergoing bending in the negative y-axis indicates that, for the top section, the upper surface undergoes tension whilst the lower surface undergoes compression. At the same time, for the bottom section, the upper surface undergoes compression whilst the lower surface undergoes tension. The following assumptions are established for this study; (a) no shearing takes place in the top and bottom sections, (b) the neutral axes of the sections remains un-deformed and (c) the wing morphing takes place within a predefined range.

The primary focus of this work is to characterise the shape sensing performance of the optical fibre sensing principle for shape sensing. This also includes understanding the fibre layout and its effectiveness on different sections of the wing.

### 3.1. Setup

**Figure 4:**
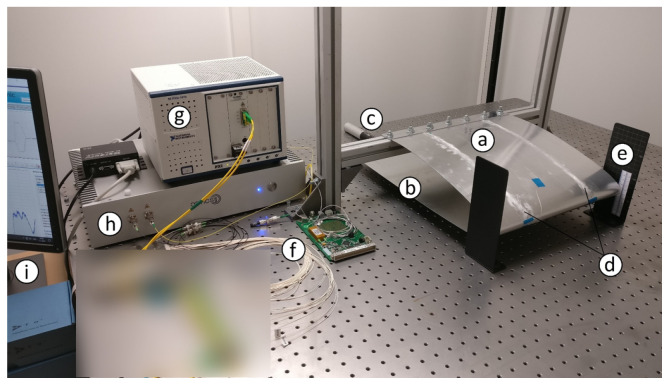
The experimental setup showing the test wing’s (**a**) fixed top surface, (**b**) sliding bottom surface, (**c**) actuator (up close in Figure 5 and Figure 6), (**d**) sensing fibres (**e**) vertical scales, (**f**) optical switch, (**g**) National Instruments interrogator, (**h**) Optics11 interrogator and the (**i**) data acquisition system.

The experimental setup is as shown in Figure 4. The test-bench was a letter ‘H’ construction made using aluminium beams (Boikon B.V., Leek, The Netherlands). The shape of the test-bench was similar to that of a rugby goal post. The two vertical uprights were 585 mm tall, 40 mm wide and were attached firmly to the optical table 590 mm apart. The horizontal crossbar of the goal post was 590 mm in width and 95 mm from the optical table. The horizontal cross bar was securely connected to the two uprights and sat snug in between them.

The top section of the wing was bolted to the top of the horizontal cross bar. It was positioned at the centre of the cross bar making it ~120 mm from each of the vertical uprights. On the other hand, the bottom section of the wing was left to hang under gravity. The only connection point of the wing to the horizontal crossbar was the top section of the wing.

An FBG spectral interrogator (National Instruments, PXIe-4844; Austin, TX, USA) measured the local strain ($\varepsilon_{1-n}$) at each of the FBG locations with a resolution of 4 pm, accuracy of 4 pm, dynamic range of 40 dB and a wavelength range from 1510 nm to 1590 nm. A second interrogator (Optics11, ZonaSens; Amsterdam, The Netherlands) measured the displacement ($\Delta L_{i-j}$) between FBG-Pairs with a resolution of 1 pm, accuracy of 1 pm, dynamic range of 160 dB and a wavelength range from 1530 nm to 1560 nm. These two interrogators operated with their own light sources that were tunable wavelength-swept lasers of class 1 M. Specious measurements were noted when light sources of both the interrogators ran together through the same fibre. This was overcome by using an optical switch (Thorlabs, OSW22-1310E; Müncher, Germany) with an automated switching algorithm operated in LabVIEW (2016) (National Instruments; Austin, TX, USA).

**Figure 5:**
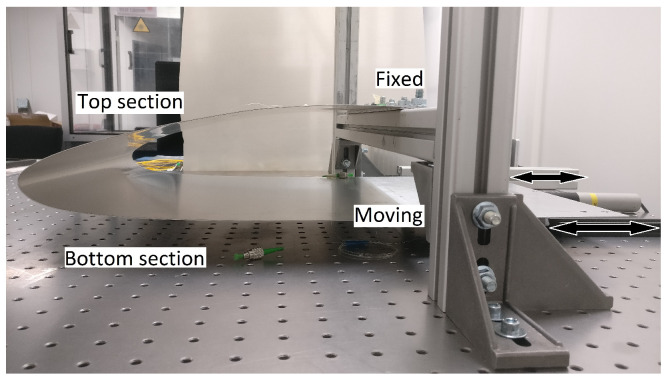
Side view of the wing attached to the support beams. The top section is fixed whilst the bottom section is allowed to slide.

In order to introduce movements to the wing, the bottom section was allowed to slide along the x-axis. Figure 5 shows the side view of the wing depicting the fixed top section and moving bottom section. For higher levels of defection involving twist, the wing resisted the actuation from one side due to how the wing was shaped. This was overcome by allowing the bottom section to move in the z-axis so as to allow the two sides of the wing to deflect in opposite (±y-axis) axes. As the resistance was higher than the force applied by the actuator, the asymmetric bending motion was done for lower deflection levels. The ranges for different movements of the wing is explained in detail in Section 4.2. As the top section was fixed, the curved part of the wing due to this motion moved up and down causing deflection in the y-axis. To facilitate this movement in a controlled manner, the bottom section of the wing had a longer chord to which an actuator was attached. The bottom section was moved using a linear actuator (PI, M-235; PI Benelux B.V., Brabant, The Netherlands) through a DC motor controller (Mercury, C-862; PI Benelux B.V., Brabant, The Netherlands) and a sliding stage.

**Figure 6:**
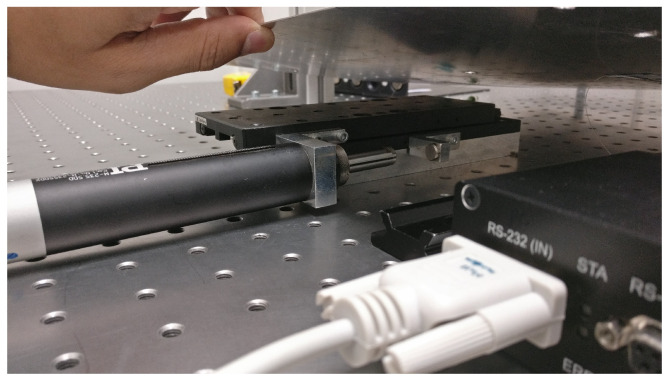
The actuation mechanism showing the actuator attached to a fixed base plate and a moveable spring loaded stage plate. The controller is in the foreground.

Figure 6 shows the actuator detached and revealed. The actuator was connected to the optical table through a fixed base plate. A spring loaded moveable stage plate was connected to the bottom section of the wing; it was capable of introducing movements with a travel range such that the wing deflected up to a maximum of 30 mm whilst allowing a rotation of 40° at the root.

### 3.2. Sensor Design

**Figure 7:**
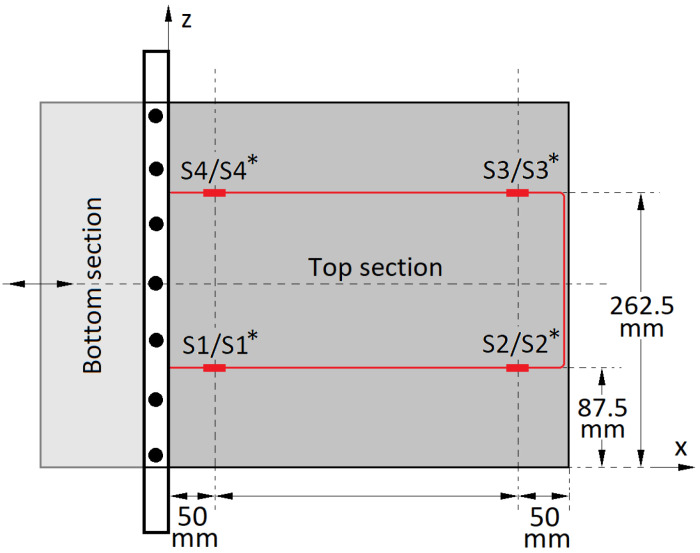
Top-view of the wing with the optical fibre running along the x-axis in the bulk of the top (and bottom) section(s). The grating sensors S1, S2, S3 and S4 are on the top section. The sensors on the bottom section are a superimposition of the top sensor positions marked with asterisks (∗); (e.g., S1 directly above S1${}^{*}$ and so on).

Figure 7 is a top-view of the wing showing the sensing fibre (in red) and its layout along with the grating sensors. Two standard single mode (SM) SMF-28e+ (Corning Incorporated, New York, NY, USA) fibres containing the grating sensors were bonded using cyanoacrylate adhesive (3M Scotch-Weld; 3M Nederland B.V., Kerkrade, The Netherland) one each on the surfaces of the top and bottom sections of the wing. For the top section, the fibre was bonded to its upper surface whilst for the bottom section the fibre was bonded to its lower surface. This was done due to ease of access as the fibre bonding process was initiated after the wing was brought to shape.

Gratings on the top section are marked as S1, S2, S3 and S4 and on the bottom section with asterisks as S1${}^{*}$, S2${}^{*}$, S3${}^{*}$ and S4${}^{*}$. The bottom surface fibre (and gratings) is a superimposition of the top surface fibre. It is to be noted that the fibre length between gratings S2/S2${}^{*}$ and S3/S3${}^{*}$ had no effect in the sensing and was hence simply partly taped and partly left free. Angled Physical Contact (FC/APC) connectors were used for all the fibre connections.

The fibre layout and sensor placement design was adopted from our earlier work [22] which specifies the importance of having sensors placed relatively close to the clamped-free (x-axis) boundary as well as having at least two sensors in the free (z-axis) boundaries of the section. Apart from this each sensor should form a pair with an adjacent sensor running chordwise, to satisfy the FBG-Pair configuration [22].

The layout of the fibres is in a U-shape pattern such that all the gratings are perpendicular to the y-axis. One end of the each fibre is connected to a switch whilst the other end is connected to an attenuator/light trap (Thorlabs, FTAPC1; Müncher, Germany) with a return loss of ⩾50 dB to reduce back reflections into the sensing fibre. Gratings S1/S1${}^{*}$ and S4/S4${}^{*}$ are at approximately 50 mm from the root and S2/S2${}^{*}$ and S3/S3${}^{*}$ at 315 mm. The gratings measure the normal strains acting in the x-axis direction.

The nominal Bragg wavelengths as specified by the manufacturer (FORC-Photonics; Moscow, Russia) of the gratings are mentioned in Table 2. As in the previous tests, gratings of length 3 mm each were chosen. To acquire best results with the interrogators, bandwidth and reflectivity levels were specified to be above 0.5 nm and 99%, respectively. The temperature sensitivity of the sensors was as low as 10 pm/°C.

## 4. Measurements

The measurement approach involved an initial calibration of the wing followed by the development of a shape sensing tool. The first step was to record baseline measurements in order to develop an experimental based wing model. A transfer function model was then used to estimate the deflection and the final shape. The experiments were carried out on different morphing cases that were brought about through the actuator. The following sub-sections describe the morphing cases used in the experimental campaign, the calibration procedure for the wing and series of tests involving arbitrary morphing to later estimate the deflections and the morphed shape.

### 4.1. Morphing Cases

**Figure 8:**
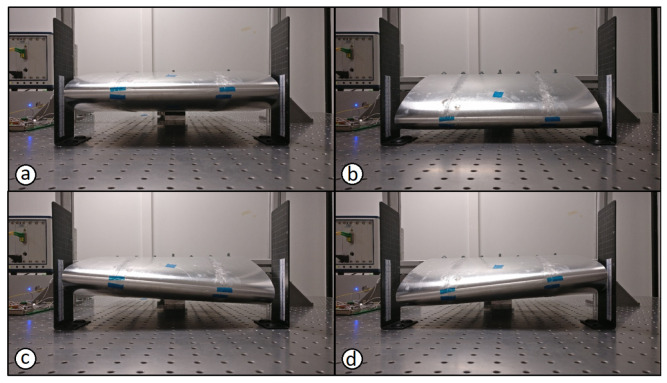
The position of the wing during (**a**) bend-up, (**b**) bend-down, (**c**) right-twist, and (**d**) left-twist morphing cases.

The wing undergoes different movements based on the actuator input. These movements are classified into four primary movements or morphing cases. The morphing cases include pure bending, torsion and a combination of bending and torsion of the wing. The morphing cases are further on termed as bend-up, bend-down, left-twist and right-twist. Their (maximum) morphing positions are as shown in Figure 8.

### 4.2. Calibration

The calibration tests were performed using the same setup as in Figure 4. The maximum and minimum ranges of deflection were mainly determined based on the actuator’s range of 50 mm. The required deflections of the wing were taken into account and the ranges were set and are shown in Table 3. This was necessary to confirm that all morphing cases could be accommodated as well as to ensure a total of 50 mm in wing tip deflection based on the actuator range.

The actuator had a push/pull force capacity up to 120 N which was sufficient to bring about all morphing scenarios. It is to be noted that the trailing edge line (along the z-axis) was assumed to be straight throughout the tests.

### 4.3. Arbitrary Morphing

The wing is morphed with unknown actuations which result in unknown deflections. They are divided into bending and twisting cases with four arbitrary cases each. These cases are termed as A, B, C and D for bending and E, F, G and H for twisting. For the bending cases, the values of $\Delta L_{1-2}$ and $\varepsilon_{1}$ should measure the same as $\Delta L_{3-4}$ and $\varepsilon_{4}$, respectively. This does not hold true for twisting. This difference is accounted for in the algorithm. A random number function was used to generate unknown actuation cases for morphing. On purpose, values of 0, mid and max wing deflections were excluded as they were already included in the calibration step. The actuation was set to input values at steps of 1 mm.

The actual wing deflections for each of the morphing cases were later measured and recorded. Table 4 and Table 5 show the right and left wing tip deflection separately for bending and twisting morphing cases, respectively.

### 4.4. Algorithm

The algorithm relies on a transfer function from the measured strain output due to actuator movement to the trailing edge tip deflection. This is attained by relating the raw FBG and FBG−Pair data to the tip deflection.

A wing-morphing → sensing-fibre-strain → trailing-edge-deflection type transfer function was used to correlate the measurement data with the wing trailing edge deflection.

Twisting of the wing is an important aspect that needs to be captured as it is part of the morphing capabilities of the a seamless wing/flap. In order to discern and distinguish the twisting motion in the wing, separate transfer functions were used for right and left twist.

Bending of a beam is a simpler motion that can be modelled using a linear interpolation approach. In this case, a quadratic interpolation was utilised to account for any non linearity.

To satisfy the condition of bending, the strain at all the points that are equidistantly measured from the root along the span should be the same. This means that an ε *FBG* measurements along with a ΔL *FBG-P* measurements should suffice. The transfer function can hence be expressed as:

(1) $$\delta =a\cdot \varepsilon_{1/4}^{2}+b\cdot \Delta L_{1-2/3-4}+c,$$

where δ is the deflection of trailing edge line (along z-axis). The coefficients *a*, *b* and *c* of the variables (ε and ΔL) are equal to −0.00012, −0.14 and 0.023, respectively, for bend down and −0.00017, 0.16 and 1.8, respectively, for bend up. The coefficients are constant for an experiment campaign and are determined by curve fitting. Based on the calibration procedure the coefficients can have a maximum deviation of ±1.3393.

The measurement data $\varepsilon_{1}$ and $\varepsilon_{4}$ particularly came into play when the positive and negative twists, respectively, were involved. To accommodate this, Equation (1) was separated into two transfer functions corresponding to positive and negative twisting.

The positive twist (right tip deflection), $\delta_{right}$, was expressed as:

(2) $$\delta_{right}=d\cdot \varepsilon_{1}^{2}+e\cdot \varepsilon_{4}+f\cdot \Delta L_{1-2}+g,$$

and the negative twist (left tip deflection), $\delta_{left}$, as:

(3) $$\delta_{left}=h\cdot \varepsilon_{1}^{2}+i\cdot \varepsilon_{4}+j\cdot \Delta L_{3-4}+k,$$

where *d* to *k* are again coefficients of the variables equal to −0.00031, 0.12, 1.5, −0.00039, −0.13 and 1.9, respectively. The coefficients are constant for a given experiment campaign and are determined by curve fitting. Based on the calibration procedure, the measurements are sensitive to the errors in the coefficients and can have a maximum deviation of ±2.408.

This separation was done in order to better capture the whole shape of the wing. Moreover, this method also covers Equation (1) that considers bending of the wing. From Equations (2) and (3) it can be proven that $\delta_{right}$ would equal $\delta_{left}$ when $\varepsilon_{1}$ = $\varepsilon_{4}$ and $\Delta L_{1-2}$ = $\Delta L_{1-2}$. This satisfies the pure bending condition.

## 5. Results

**Figure 9:**
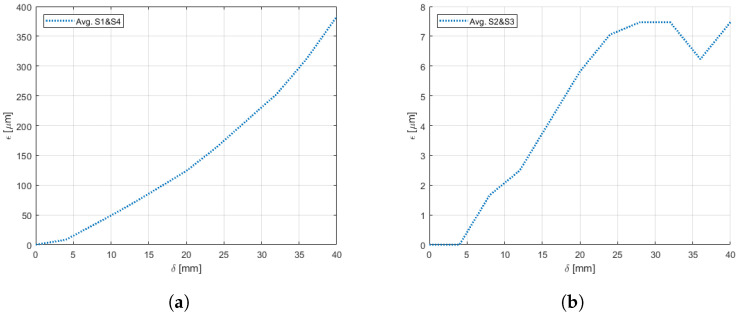
Strain trend in the top section of the wing through sensors (**a**) S1 and S4 and (**b**) S2 and S3, undergoing bend-down with respect to deflection due to morphing. Separate figures are used due to the large variation in scales.

**Figure 10:**
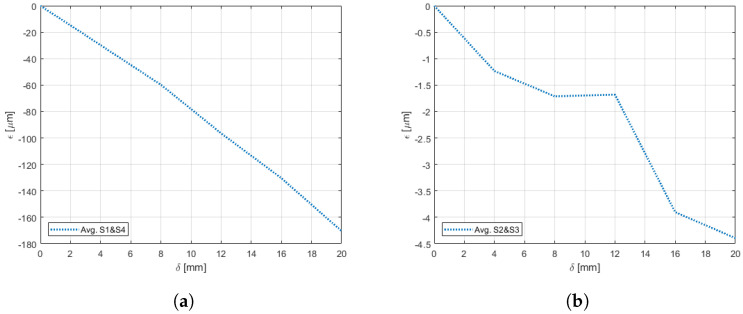
Strain trend in the top section of the wing through sensors (**a**) S1 and S4 and (**b**) S2 and S3, undergoing bend-up with respect to deflection due to morphing. Separate figures are used due to the large variation in scales.

**Figure 11:**
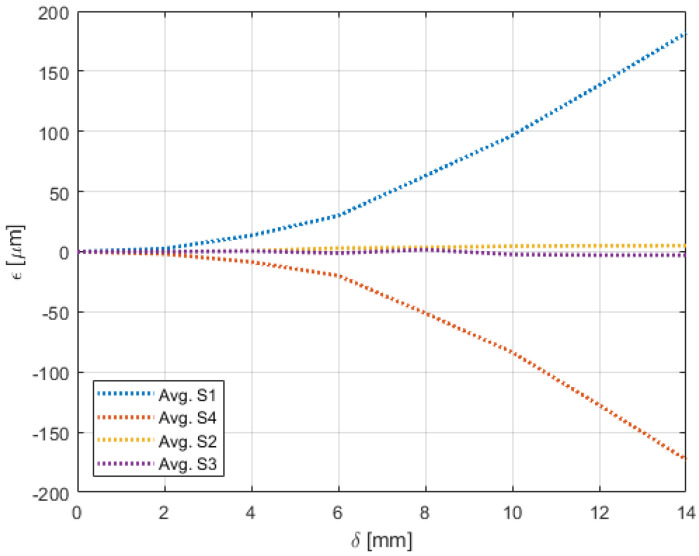
Strain trend in the top section of the wing undergoing twist with respect to deflection due to morphing.

**Figure 12:**
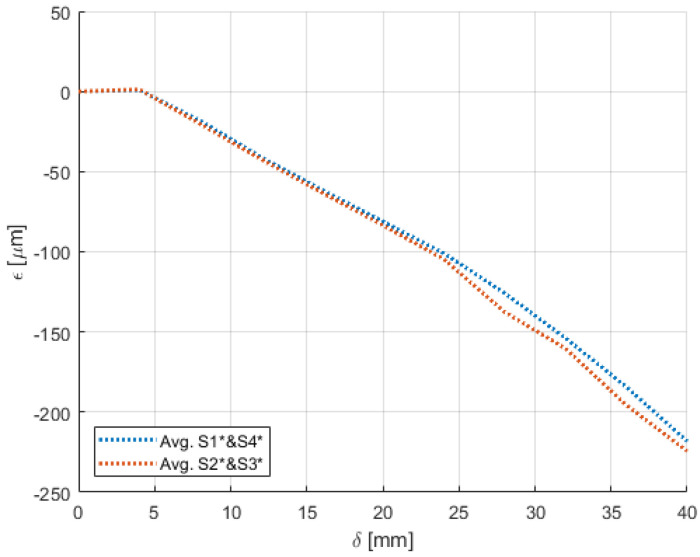
Strain trend in the bottom section of the wing undergoing bend-down with respect to deflection due to morphing.

**Figure 13:**
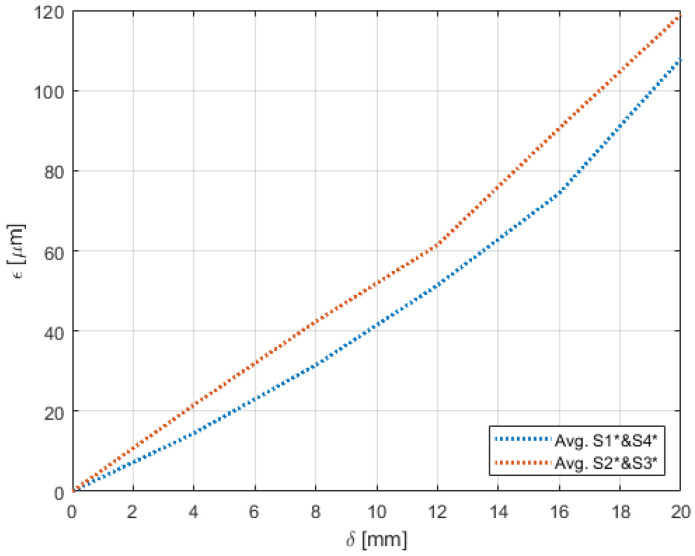
Strain trend in the bottom section of the wing undergoing bend-up with respect to deflection due to morphing.

**Figure 14:**
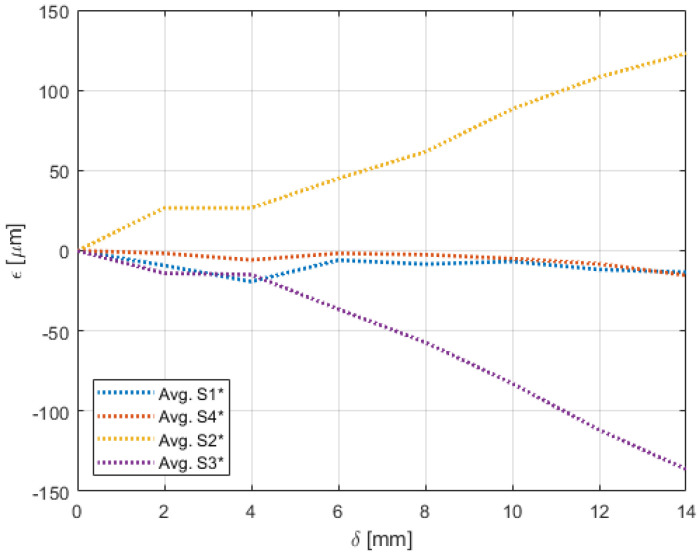
Strain trend in the bottom section of the wing undergoing twist with respect to deflection due to morphing.

Experiments pertaining to the four morphing cases were carried out following the procedure elaborated in Section 4. Figure 9, Figure 10, Figure 11, Figure 12, Figure 13 and Figure 14 show the strain output of the sensors for their respective deflection for the top and bottom section, respectively. The positive and negative values imply that the fibres (and in turn the sensors) undergo tension and compression, respectively. Theoretically, for a bend up (and bend down), sensor pairs S1 and S4 should be identical (in magnitude and trend) and so should sensors pairs S2 and S3. On the other hand, in case of twist these pairs should also show identical readings but with opposite polarity (Figure 11 and Figure 14). The slight offset between the two could mean one of the following; (a) one side is being bent more than the other or (b) the grating is closer to the root than the other grating causing it to experience higher strain. In twist, this disparity increases because the difference between the pairs themselves is added to the deflection error. Generally, the offset due to the errors resulting from improper fibre positioning and/or sensor position is already corrected at the strain calculation level. In this study, the focus of study was mainly on the trend they followed and hence the average of the pairs are shown. Comparisons of the estimated deflections with the actual deflections are tabulated in the following section.

For the top section, a clear distinction between the sensors near the root (S1 and S4) and at tip (S2 and S3) was noticed for the bend cases (Figure 9 and Figure 10). Although the maximum deflection was higher for bend down than bend up, the trend line was similar for both the cases. Moreover, although the twist case (Figure 11) showed low strains for small deflections, these also had similar trend as the bend cases.

On the other hand, the sensors on the bottom section had a different behaviour for both the bend and twist cases. Since the configuration of the bottom section was not similar to a cantilever setup, the sensors near the root (S1 and S4) and at tip (S2 and S3) did not have a clear distinction for bending (Figure 12 and Figure 13). Unlike the top section, the bottom section experienced a sliding motion in addition to the bending motion. In the bend down case, for the first few mm the sliding motion was dominant and hence no strain was experienced. This explains the flat line from 0 mm to 4 mm.

The biggest difference was seen in the twist case (Figure 14) where a messy pattern was observed. Again, this was attributed to the bottom section undergoing a combination of sliding and bending. In addition to that, as explained in Section 3.1, the bottom section also had movements in the z-axis that introduced strain that are not along the axis of the fibre.

### Arbitrary Morphing

Eight arbitrary morphing cases were tested and their estimated deflections were compared with the actual deflections. The errors in the estimated deflections for morphing cases A to D are as shown in Table 6 and have a maximum error of 0.45 mm. Table 7 and Table 8 show the estimated deflections for morphing cases E to H and these have a maximum error of 0.7 mm.

## 6. Discussion

Estimation of the deflection due to arbitrary morphing that include bend up, bend down and twist of a morphing wing mockup is demonstrated. The wing morphing was facilitated by an actuator that was attached to the sliding bottom section of the wing. The aim of the study was also to understand the difference between placing the sensing fibre at two locations on the wing and to conclude on the most desirable location.

The measurements were recorded through two optical fibres bonded on each to the top and bottom sections of the wing. They were single-core Single Mode (SM) fibres containing two pairs of fibre Bragg gratings each. The experiments were conducted within a controlled laboratory environment where the temperature variations were considered low. The temperature was assumed to be constant throughout the experiment.

On any morphing sequence, the change in shape of the wing was measured in terms of strain on the fibres. The sensors placed closer to the clamp pick up local strain whilst the sensors on the free end do not measure as this end has a free boundary condition. The sensors at the free end were used to measure the displacement along the chord of the wing as they formed sensor pairs with the sensors near the clamp. Using the reference measurements as baseline data the algorithm predicted the deflection of the trailing edge. The trailing edge line (along the z-axis) was assumed to be straight throughout the experiment. Due to the thickness of the material being negligible compared to other dimensions the shear strains on the wing are not considered here.

The wing had a natural bend due to gravity and the reference zero strain values were measured after the wing was suspended. On being morphed, two local strain readings were obtained near the wing root of the top section. As the other sides of the top section were not fixed, the maximum strain was always near the clamp (similar to a cantilever case). The other set of measurements were the two strain components along the chord of the wing that were similar during bending and different during twisting.

The trailing edge was displaced along the y-axis caused by the movement of the bottom section. The trailing edge had a maximum dip of 40 mm that gradually reduced to 0 towards the clamped region. A symmetric behaviour was also noticed when the sliding (bottom) section was displaced (to a maximum of 20 mm) but in the positive x-direction. To distinguish these two cases, the distinction between compression and tension induced strain was identified at the interrogator level by the sign of the strain. This was also crucial because it helped in identifying the positive and negative wing movement which was essential for shape sensing. This also confirms the placement of the fibres on each surface. For the top section the fibre was placed on its upper surface such that during a bend-down the fibre experienced tension and vice versa. It was noticed that the tip (trailing edge line) no longer had the highest deflection point in a twist configuration. The actuator had to put in extra effort for twist and the maximum dip achieved for the trailing edge deflection was ±14 mm but still following symmetry.

On the whole, the shape of the morphing wing was estimated within 1 mm of the trailing edge deflection. For lower deflections up to ~10 mm the estimation was almost linear. The non linearity gradually increased for higher deflections which also signified the wing leaving the cantilever domain. For deflections higher than 10 mm, for small actuator inputs the trailing edge deflection was higher. This was due to the surface experiencing a slight curving effect.

Hysteresis tests were carried out by gradually deflecting the wing from 0 mm all the way to the higher limits and back to 0 mm. The hysteresis detection limit was set as 1 mm. For all the trials the wing showed no hysteresis when measured on the vertical scale. Moreover, the actuator was measured to have a position accuracy of 0.0002 mm whose effect on the estimation was negligible.

The fibre on the top section on comparison with the bottom section gave a better distinction between the sensors near the clamped end and the free end. Moreover, the twist case for the bottom section had a messy trend as compared to a more discernible and smooth trend for the top section. The presence of a sensing fibre on the top section alone would hence suffice for the deflection estimation.

A limitation here is that bend up/down and twist readings were measured separately. Although the aluminium wing construction was flexible, it resisted the movement from one side during a twist inducing morphing, making the twisting motion difficult. For example, the actuation displacement required for an *x* mm bend down was not equivalent to an *x* mm twist. To tackle this, a mechanism would be required to isolate both the sides of the wing so that they could operate similar to a twist. In other words, the movement for a one-sided twist should be equivalent to the movement of a one-sided bend. A way to realise this would be to consider the (top and bottom) section of the wing as two individual wings joined together. This could be visualised from Figure 3 by drawing a line along the x-axis in the middle of the wing considering it to divide the wing into two halves. Each half would have its dedicated actuator. However, this would create issues such as gaps and an uneven surface. An alternative would be to have a region in between them that is strong yet flexible enough to overcome the issues mentioned above, that is, to have a smooth surface with no gaps and hence no/low resistance when the two wing sections are morphed independently.

## 7. Conclusions

The aim of this work was to demonstrate shape sensing by estimating the trailing edge deflection due to arbitrary morphing movements on wing sections. Furthermore, different fibre locations were compared and a recommendation on the most desirable location to place the sensing fibre was made.

The fibre on the top section had better capabilities of monitoring bending and twisting of the wing. For small deflections the estimation resembled a wide cantilever beam. The width was not a determining factor in the estimation as the deflection was independent of the width of the wing. For higher deflections non-linearity was noticed which was attributed to the curving of the wing surfaces. Based on the calibration data a transfer function model was used to estimate the trailing edge deflections. Since a better distinction was possible using just the sensor readings on the top section of the wing, it was concluded that having the top fibre alone was sufficient. The multimodal sensing approach yielded a maximum deflection sensing error of 5.8% for a 347 × 350 mm wing section.

Although the model has been developed around the morphing trailing edge, this approach can potentially be used to model any morphing structure, regardless of its material provided they exhibit elastic properties. Furthermore, this study opens the design space for future designs and design iterations of the morphing wing. As materials properties are not a constraint, with proper calibration, full composite wing structures can also be studied.

A study on the fibre placement and recommendations for efficient monitoring is also included. The analysis yielded a maximum deflection sensing error of 0.7 mm for a 347 × 350 mm wing section.

## Figures and Tables

**Table 1:** Material and geometric properties of the wing.

Property	Variable	Value
Material		Aluminium
Chord (top)	$c_{t}$	343 mm
Span (top)	$s_{t}$	350 mm
Chord (bottom)	$c_{b}$	570 mm (343 + 227)
Span (bottom)	$s_{b}$	350 mm
Thickness	*t*	1 mm

**Table 2:** Properties of the fibre Bragg grating sensor pairs S1/S1${}^{*}$, S2/S2${}^{*}$, S3/S3${}^{*}$ and S4/S4${}^{*}$ (FORC-Photonics; Moscow, Russia). Unlike other sensor pairs, S3/S3${}^{*}$ had different wavelengths and are mentioned separately.

Property	*S*1/*S*1${}^{*}$	*S*2/*S*2${}^{*}$	*S*3	*S*3${}^{*}$	*S*4/*S*4${}^{*}$
Wavelength	1529.3 nm	1539.3 nm	1549.4 nm	1549.1 nm	1559.4 nm
Bandwidth	← 0.5 ± 0.1 nm →				
Reflectivity	←⩾99% →				
Temp. sensitivity	← 10 pm/°C →				

**Table 3:** Allowable movement ranges specified and set by the calibration procedure to adhere to the structural design limits. This pertains to the actuator (x-axis) and the deflection of the wing tip (z-axis).

Parameter	Bend-Up	Bend-Down	Twist
Actuator (x-axis)	−25 mm	+25 mm	±15 mm
Tip deflection (z-axis)	−20 mm	+40 mm	±15 mm

**Table 4:** Actual deflections of the wing during bending due to arbitrary morphing A to D.

Morphing	$\delta_{\mathrm{right}}$ (mm)	$\delta_{\mathrm{left}}$ (mm)
A	−7	−7
B	−16	−16
C	13	13
D	28	28

**Table 5:** Actual deflections of the wing during twisting due to arbitrary morphing E to H.

Morphing	$\delta_{\mathrm{right}}$ (mm)	$\delta_{\mathrm{left}}$ (mm)
E	9	−9
F	12	−12
G	−3	3
H	−13	13

**Table 6:** Model estimated deflections of the wing during bending due to arbitrary morphing A to D.

Morphing	Actual δ (mm)	Estimated δ (mm)	Maximum Error (mm)	Error (%)
A	−7	−6.9	−0.1	1.4
B	−16	−15.9	−0.1	0.6
C	13	12.6	0.4	3.1
D	28	28.6	−0.6	2.1

**Table 7:** Model estimated right deflections of the wing during twisting due to arbitrary morphing E to H.

Morphing	Actual $\delta_{\mathrm{right}}$ (mm)	Estimated $\delta_{\mathrm{right}}$ (mm)	Maximum Error (mm)	Error (%)
E	9	9.3	−0.3	3.3
F	12	12.5	−0.5	4.1
G	−3	−2.5	−0.5	16.6
H	−13	−13.2	0.2	1.5

**Table 8:** Model estimated left deflections of the wing during twisting due to arbitrary morphing E to H.

Morphing	Actual $\delta_{\mathrm{left}}$ (mm)	Estimated $\delta_{\mathrm{left}}$ (mm)	Maximum Error (mm)	Error (%)
E	−9	−9.2	0.2	2.2
F	−12	−12.7	0.7	5.8
G	3	2.6	0.4	13.3
H	13	13.3	−0.3	2.3

## Data Availability

The data pertaining to this work are available on request.
